# Entwicklung von Antibiotikaanwendung und -resistenz in der Tierhaltung 2014–2024 am Beispiel von Mastputen und -hühnern sowie Auswirkungen auf den Menschen

**DOI:** 10.1007/s00103-026-04227-5

**Published:** 2026-04-02

**Authors:** Matthias Flor, Bernd-Alois Tenhagen

**Affiliations:** Fachgruppe Epidemiologie, Zoonosen und Antibiotikaresistenz, Abteilung Biologische Sicherheit, Bundesinstitut für Risikobewertung, Max-Dohrn-Str. 8–10, 10589 Berlin, Deutschland

**Keywords:** Therapiehäufigkeit, Zoonosenmonitoring, Antibiotikaminimierungskonzept, *Escherichia coli*, *Campylobacter *spp., Treatment frequency, Zoonosis monitoring, Antibiotics minimization concept, *Escherichia coli*, *Campylobacter *spp.

## Abstract

**Einleitung:**

In der Nutztierhaltung werden Antibiotika oft zur Behandlung von Tiergruppen eingesetzt, z. B. bei Geflügel. Mit der 16. Novelle des Arzneimittelgesetzes wurde 2014 in Deutschland das Antibiotikaminimierungskonzept eingeführt. In der Folge reduzierte sich die Therapiehäufigkeit in vielen Tierpopulationen. Ziel dieser Studie ist es, die Entwicklung des Antibiotikaeinsatzes und der Resistenz in den Populationen von Mastputen und -hühnern und die Auswirkungen auf die Resistenzsituation in der Humanmedizin darzustellen.

**Methoden:**

Die Therapiehäufigkeiten bei Mastputen und -hühnern wurden für 2014–2024 mit den aus dem Zoonosen-Monitoring stammenden Resistenzraten von *Escherichia (E.) coli, Campylobacter (C.) coli* und *C. jejuni* gegenüber Antibiotika verglichen und anhand der Joint Interagency Antimicrobial Consumption and Resistance Analysis (JIACRA)-Arbeiten in Bezug zur Resistenzsituation beim Menschen gesetzt.

**Ergebnisse:**

Die Therapiehäufigkeit sank bei Mastputen von 61,9 auf 45,6 Tage (−26 %), stieg aber bei Masthühnern von 41,5 auf 45,9 Tage (+11 %). Der Einsatz kritischer Antibiotika wurde in beiden Populationen stark reduziert. Bei Mastputen sanken die Resistenzraten von *E. coli* signifikant, wohingegen bei Masthühnern teilweise Anstiege beobachtet wurden. Die Resistenz von *C. jejuni* gegenüber Ciprofloxacin stieg trotz reduziertem Antibiotikaeinsatz. Die JIACRA-Berichte zeigen, dass die Resistenzentwicklung bei *E. coli* von Tieren und Menschen mit dem Antibiotikaeinsatz in der jeweiligen Population verbunden ist, während bei *Campylobacter* eine direkte Beziehung zwischen tierischen und humanen Resistenzraten besteht.

**Diskussion:**

Die Ergebnisse verdeutlichen die Notwendigkeit gezielter Maßnahmen zur weiteren Reduktion des Antibiotikaeinsatzes und deuten auf komplexe Dynamiken der Resistenzentwicklung hin.

## Abstract (en)

**Introduction:**

In livestock, antibiotics are often used to treat groups of animals, such as poultry. With the 16th amendment of the Medicinal Products Act, the antibiotic minimization concept was introduced in Germany in 2014. As a result, treatment frequencies were reduced in many animal populations. The aim of this study is to present the development of antibiotic use and resistance in the populations of fattening turkeys and broiler chickens as well as the effects on the resistance situation in human medicine.

**Methods:**

The treatment frequencies in fattening turkeys and broiler chickens for 2014–2024 were compared with data from zoonosis monitoring, namely the resistance rates of *Escherichia (E.) coli*, *Campylobacter (C.) coli*, and *C. jejuni* against antibiotics, and contextualized in relation to the human resistance situation based on Joint Interagency Antimicrobial Consumption and Resistance Analysis (JIACRA) work.

**Results:**

The treatment frequency decreased from 60.9 to 45.6 days (−26%) in fattening turkeys but increased from 41.5 to 45.9 days (+11%) in broiler chickens. The use of critically important antibiotics has been greatly reduced in both populations. In fattening turkeys, the resistance rates of *E. coli* decreased significantly, whereas in broiler chickens, some increases were observed. Resistance of *C. jejuni* to ciprofloxacin increased despite reduced antibiotic use. The JIACRA reports show that the development of resistance in *E. coli* from animals and humans is associated with the use of antibiotics in the respective population, whereas in *Campylobacter* there is a direct relationship between animal and human resistance rates.

**Discussion:**

The results illustrate the need for targeted measures to further reduce the use of antibiotics and point to complex dynamics in resistance development.

## Einleitung

Antibiotische Substanzen werden nicht nur beim Menschen, sondern auch bei Nutztieren und anderen Haustieren zur Therapie von Erkrankungen eingesetzt. Dabei unterscheidet sich der Einsatz bei den Nutztieren grundsätzlich von dem bei den anderen Haustieren, weil der Einsatz beim Nutztier neben der Therapie des Einzeltiers immer auch den Aspekt des Schutzes der Gesundheit der anderen Tiere im Tierbestand hat. Ein weiterer wesentlicher Aspekt ist, dass häufig Gruppen von zusammen gehaltenen Tieren behandelt werden, weil eine Individualtherapie aufgrund der großen Zahl der gehaltenen Tiere und ihrer bedingten Zugänglichkeit oft nicht praktikabel ist.

Bei der Therapie von Tiergruppen mischen sich wohlgemerkt immer die Therapie des erkrankten Tieres und die Behandlung von Tieren, die ggf. noch nicht klinisch erkrankt, aber mit einer hohen Wahrscheinlichkeit einer hohen Infektionsdosis dieses Erregers ausgesetzt sind, sodass es darum geht, die klinische Erkrankung gar nicht erst ausbrechen zu lassen. Insbesondere bei Schweinen und beim Geflügel steht die Gruppentherapie im Vordergrund, weil bei der Therapie von Einzeltieren, z. B. in Herden von Masthühnern, das Aufsuchen und Fangen der individuellen Tiere nicht nur sehr aufwendig wäre, sondern auch eine erhebliche Unruhe in die Herde bringen würde, was bei einer Nutzenabwägung den Sinn der Therapie infrage stellen würde.

Seit im Jahr 2011 erstmals die in Deutschland an Tierärztinnen und Tierärzte verkauften Mengen antimikrobieller Tierarzneimittel erfasst und veröffentlicht wurden, gab es erhebliche Anstrengungen, diese Menge deutlich zu reduzieren. Ihren rechtlichen Niederschlag fanden diese Bemühungen 2014 in der 16. Novelle des Arzneimittelgesetzes, mit der zur Mast gehaltene Tiere unterschiedlicher Tierarten einem Benchmarkingsystem im Hinblick auf den Einsatz antimikrobieller Tierarzneimittel unterworfen wurden. In der Folge ging die Therapiehäufigkeit in vielen dieser Populationen deutlich zurück [1]. Einen umfassenden Überblick über die Entwicklung bieten die jährlich vom Bundesinstitut für Risikobewertung (BfR) herausgegebenen Berichte zum Antibiotikaeinsatz in der Nutztierhaltung, die online verfügbar sind [2].

Im ersten Teil dieses Beitrags wird es um die Entwicklung des Antibiotikaeinsatzes in Deutschland sowie eine grobe Abschätzung gehen, ob und in welchem Umfang die Reduktionsmaßnahmen zu einer Verbesserung der Resistenzsituation bei Bakterien aus der Lebensmittelkette geführt haben. Dabei wird dies exemplarisch für die beiden Sektoren Masthühner und Mastputen sowie für *Escherichia coli* und *Campylobacter* spp. dargestellt, weil anhand dieser beiden Parameter (Tierpopulation und Bakterienspezies) Unterschiede aufgezeigt werden können, die die Komplexität der Fragestellung bereits auf dieser Ebene beschreiben.

Im zweiten Teil werden wir dann der Frage nachgehen, ob und inwiefern diese Veränderungen zu einer etwaigen Verbesserung der Resistenzsituation in der Humanmedizin beigetragen haben könnten. Hierfür wechseln wir auf die europäische Ebene und verwenden als Grundlage vor allem die von einer gemeinsamen Arbeitsgruppe der Europäischen Behörde für Lebensmittelsicherheit (EFSA), der Europäischen Arzneimittel-Agentur (EMA) und dem Europäischen Zentrum für die Prävention und die Kontrolle von Krankheiten (ECDC) vorgelegten Berichte zur „Joint Interagency Antimicrobial Consumption and Resistance Analysis“ (JIACRA; [3]). Diese Berichte betrachten anhand der auf europäischer Ebene vorliegenden Daten zum Antibiotikaverbrauch und zur Antibiotikaresistenz das Verhältnis von Antibiotikaanwendung und -resistenz bei Menschen und Tieren und die dazwischen bestehenden Beziehungen. Auch hier werden wir vor allem die Komplexität der Problematik darstellen, weil es eine einfache und allgemeingültige Antwort auf die Frage nicht gibt.

## Methoden

### Antibiotikaminimierungskonzept: Antibiotikaeinsatz bei Nutztieren in Deutschland

Im ersten Teil werden die im Rahmen des Antibiotikaminimierungskonzeptes erfassten und vom BfR plausibilisierten und ausgewerteten Antibiotikaeinsatzdaten verwendet. Das Antibiotikaminimierungskonzept wurde mit der 16. Novelle des Arzneimittelgesetzes im Jahr 2014 für Masttiere eingeführt [4] und seitdem mehrfach weiterentwickelt [5–7]. Es definiert ein Benchmarkingsystem auf Betriebsebene, das für verschiedene Nutzungsarten bei Rindern, Schweinen, Hühnern und Puten gilt. Betriebe derselben Nutzungsart werden auf Basis ihrer betrieblichen Therapiehäufigkeit (siehe Infobox 1) mit antibiotisch wirksamen Substanzen in halbjährlichem Rhythmus miteinander verglichen und anhand von 2 Kennzahlen eingruppiert. Betriebe, deren Therapiehäufigkeit oberhalb von Kennzahl 1 liegt, sollen unter tierärztlicher Beratung nach Maßnahmen suchen, um ihren Antibiotikaeinsatz zu reduzieren, und Betriebe oberhalb von Kennzahl 2 sind verpflichtet, entsprechende Maßnahmenpläne den Landesbehörden vorzulegen. Als Maß für den Antibiotikaeinsatz misst die halbjährliche betriebliche Therapiehäufigkeit (TH),

1 $$\begin{aligned} &\mathrm{TH}=\\&\frac{\left\{\begin{array}{c}\sum \big((\text{Anzahl behandelter Tiere})\\\times (\text{Anzahl Wirktage})\\\times (\text{Anzahl Wirkstoffe})\\\times (\mathrm{Wichtungsfaktor})\big)\end{array}\right\}}{\left\{\begin{array}{c}\text{Durchschnittliche Anzahl}\\ \text{gehaltener Tiere}\end{array}\right\}}, \end{aligned}$$

an wie vielen Tagen des Halbjahres ein durchschnittlich im Betrieb gehaltenes Tier einer Nutzungsart antibiotisch behandelt wurde. Ein „durchschnittlich im Betrieb gehaltenes Tier“ bzw. die durchschnittliche Anzahl gehaltener Tiere im Nenner von Gl. 1 sind dabei rechnerische Größen, bei denen implizit sowohl Betriebsgröße als auch Haltungsdauer der Tiere und Leerstandzeiten berücksichtigt werden, sodass unterschiedliche Betriebe miteinander verglichen werden können. Die Summation im Zähler von Gl. 1 erfolgt über alle antibiotischen Anwendungen, die in einem Betrieb in der jeweiligen Nutzungsart innerhalb des Halbjahres stattgefunden haben. Die Wirktage entsprechen bei klassischen Präparaten den Behandlungstagen. Bei sogenannten Long-Acting-Präparaten, bei denen durch einmalige Gabe ein Wirkspiegel über mehrere Tage erzeugt wird, und bei One-Shot-Präparaten, die über eine höhere Dosierung einen nachhaltigen Effekt erzielen, werden die Wirktage nach einer im Tierarzneimittelgesetz vorgeschriebenen Formel festgelegt [6].

Der Wichtungsfaktor in Gl. 1 wurde mit der Anpassung des Tierarzneimittelgesetzes im Jahr 2023 für Cephalosporine der 3. und 4. Generation, Fluorchinolone sowie das Polypeptidantibiotikum Colistin eingeführt.^1^ Diese kritischen Wirkstoffe werden von der Antimicrobial Advice ad hoc Expert Group (AMEG) der EMA in der Kategorie B und somit als von kritischer Bedeutung für die Humanmedizin eingestuft (siehe Infobox 2 und [8]). Im Zuge derselben Gesetzesänderung wurden auch die Nutzungsarten neu zugeschnitten. Das Bundesinstitut für Risikobewertung berechnet in seinen Berichten [9–13] neben den betrieblichen Therapiehäufigkeiten außerdem sogenannte populationsweite Therapiehäufigkeiten (siehe Infobox 1). Diese spiegeln den durchschnittlichen Antibiotikaeinsatz in der gesamten Tierpopulation der Nutzungsart wider. Dies erlaubt den Vergleich der Nutzungsarten miteinander. Die populationsweiten Therapiehäufigkeiten werden ebenfalls gemäß Gl. 1 berechnet, beziehen sich jedoch auf ein gesamtes Jahr. Im Zähler werden bei der populationsweiten Therapiehäufigkeit die antibiotischen Anwendungen aller Betriebe der jeweiligen Nutzungsart berücksichtigt und im Nenner entsprechend die in allen Betrieben gehaltenen Tiere der Nutzungsart. Das BfR berechnet populationsweite Therapiehäufigkeiten auch für die einzelnen Wirkstoffklassen, sodass auch die Häufigkeit der Anwendung kritischer Wirkstoffe analysiert werden kann. Für das Antibiotikaminimierungskonzept wird jeglicher Einsatz von Antibiotika unter Angabe von Name und Menge des verwendeten Tierarzneimittels, der Nutzungsart und Zahl der behandelten Tiere und der Zahl der Behandlungstage in einer Datenbank erfasst, allerdings ohne Angabe der Indikation für die Behandlung.

Als exemplarische Nutzungsarten wurden für die vorliegende Arbeit Mastputen und Masthühner herangezogen, für die auch aus dem Zoonosen-Monitoring stammende Resistenzdaten vorliegen (siehe folgender Abschnitt). Um eine Vergleichbarkeit über die Zeit zu gewährleisten, wurden die populationsweiten Therapiehäufigkeiten für den gesamten untersuchten Zeitraum von 2014 bis 2024 ohne Wichtungsfaktoren verwendet. Diese Daten finden sich in den vom BfR publizierten Berichten [2].

### Zoonosen-Monitoring: Antibiotikaresistenz von *E. coli* und *Campylobacter* von Schlachttieren

Den Daten zum Antibiotikaeinsatz gegenübergestellt werden Resistenzdaten, die aus dem Zoonosen-Monitoring stammen [14]. Dieses wird in Deutschland auf Grundlage der Allgemeinen Verwaltungsvorschrift über die Erfassung, Auswertung und Veröffentlichung von Daten über das Auftreten von Zoonosen und Zoonoseerregern entlang der Lebensmittelkette (AVV Zoonosen Lebensmittelkette) durchgeführt, die wiederum auf der europäischen Richtlinie 2003/99/EG zur Überwachung von Zoonosen und Zoonoseerregern basiert. Darin ist ein gemeinschaftliches Verfahren zur Überwachung von Zoonosen geregelt. Die Mitgliedstaaten der EU sind dazu verpflichtet, repräsentative und vergleichbare Daten über das Auftreten von Zoonoseerregern in Futtermitteln, lebenden Tieren und Lebensmitteln sowie über die in diesen Mikroorganismen auftretenden Resistenzen gegenüber festgelegten Panels an antibiotischen Wirkstoffen zu erfassen, auszuwerten und darüber zu berichten. In Deutschland führen die Länder die Probenahmen durch, analysieren die Proben in eigenen Labors und melden ihre Befunde an das Bundesamt für Verbraucherschutz und Lebensmittelsicherheit (BVL), das die Daten für alle Bundesländer sammelt und auswertet. Zudem werden die gewonnenen Isolate an die Nationalen Referenzlaboratorien (NRL) am BfR übersandt, um dort genauer typisiert und auf Resistenz gegen antimikrobielle Substanzen untersucht zu werden. Zu den überwachten Mikroorganismen gehören *Salmonella* spp., *Campylobacter (C.)* spp., kommensale *Escherichia (E.) coli*, Extended-Spektrum-Beta-Laktamase und AmpC-Beta-Laktamase-bildende *E. coli* sowie Carbapenemase-bildende *E. coli*, die von Rindern, Schweinen und Geflügel sowie aus den von diesen Tieren gewonnenen Lebensmitteln stammen. Dabei wird jeweils in geraden Jahren die Lebensmittelkette Geflügel und in ungeraden Jahren die Lebensmittelkette Rind/Schwein beprobt.

In der vorliegenden Arbeit beschränken wir uns auf die Darstellung der Entwicklung von Antibiotikaresistenzen bei kommensalen *E. coli* sowie bei den Zoonoseerregern *C. coli* und *C. jejuni*, isoliert aus dem Blinddarminhalt von Mastputen und Masthühnern bei der Schlachtung jeweils in geraden Jahren von 2014 bis 2024. Um die Entwicklungstendenz der Resistenzraten gegenüber den verschiedenen antibiotischen Substanzen zu analysieren, wurden jeweils univariate logistische Regressionen mit dem Probenahmejahr als unabhängige Variable durchgeführt. Die Daten können den vom BVL veröffentlichten Bund-Länder-Berichten zum Zoonosen-Monitoring oder dem BfR-Datenportal ZooNotify entnommen werden [14, 15].

### Beschreibung des Vorgehens im JIACRA-Bericht

Die JIACRA-Arbeitsgruppe von EFSA, EMA und ECDC verwendet Daten, die von den Mitgliedstaaten an die Europäischen Behörden gemeldet werden und Informationen zum Einsatz von Antibiotika bei Mensch und Tier sowie zur Antibiotikaresistenz bestimmter Mikroorganismen enthalten [3]. Die Beziehung zwischen dem Einsatz von Antibiotika in den beiden Populationen (Mensch und Tier) und der Resistenzsituation bei Bakterien aus diesen Populationen – insbesondere beim Menschen – wurde anhand von univariaten und multivariaten Analysen untersucht. Die multivariaten Analysen wurden dabei mit der Methode des Partial Least Squares Path Modelling (PLS-PM) durchgeführt. Im jüngsten Bericht wurde darüber hinaus auch der zeitliche Trend von Verbrauch und Resistenz innerhalb der Populationen untersucht. Für Details zu den Analysen wird auf die entsprechenden Berichte der Arbeitsgruppe verwiesen [16–19].

## Ergebnisse

### Entwicklung von Antibiotikaeinsatz und Resistenzraten bei Mastputen und Masthühnern

**Abb. 1:**
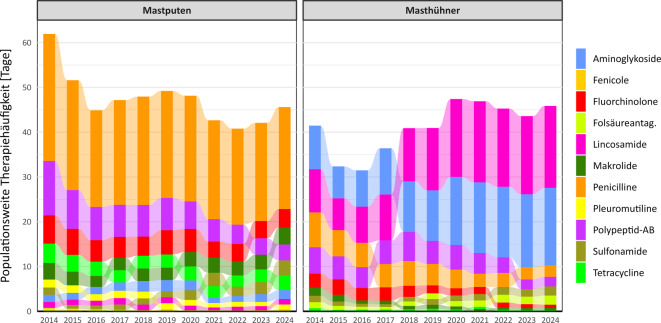
Entwicklung der populationsweiten Therapiehäufigkeit je Wirkstoffklasse bei Mastputen und Masthühnern, 2014 bis 2024. Die Wirkstoffklassen sind in jedem Jahr absteigend von höchster zu niedrigster Therapiehäufigkeit sortiert, sodass Verschiebungen bei der relativen Behandlungshäufigkeit sichtbar werden. Berechnung der Therapiehäufigkeiten ohne Wichtungsfaktoren. Für das Jahr 2014 wurden die Werte aus dem 2. Halbjahr verdoppelt. *AB* Antibiotika, *Folsäureantag.* Folsäureantagonisten

**Abb. 2:**
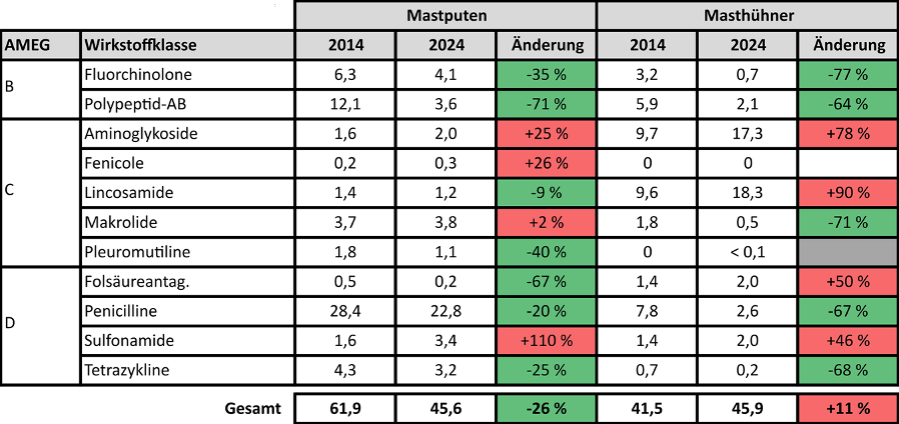
Populationsweite Therapiehäufigkeit in Tagen je Wirkstoffklasse bei Mastputen und Masthühnern für die Jahre 2014 und 2024 sowie prozentuale Änderung. Farbkodierung in den Spalten „Änderung“: *Grün* – Abnahme; *rot* – Zunahme; *grau* – ohne Angabe (prozentuale Änderung irreführend wegen kleiner Absolutwerte); *weiß* – kein Einsatz. *AB* Antibiotika, *AMEG* Kategorien der „Antimicrobial Advice ad hoc Expert Group“ der Europäischen Arzneimittel-Agentur (EMA), *Folsäureantag*. Folsäureantagonisten

Für den Zeitraum 2014 bis 2024 ist in Abb. 1 für Mastputen und Masthühner die Entwicklung der populationsweiten Therapiehäufigkeit insgesamt und je Wirkstoffklasse dargestellt. Zudem enthält Abb. 2 die dazugehörigen Zahlenwerte für die Jahre 2014 und 2024 sowie die prozentualen Änderungen der populationsweiten Therapiehäufigkeiten je Wirkstoffklasse von 2014 zu 2024. In beiden Nutzungsarten war und ist der Antibiotikaeinsatz hoch. Für Mastputen waren im Jahr 2014 insgesamt 61,9 Tage mit antibiotischer Behandlung je durchschnittlich gehaltenem Tier und Jahr zu verzeichnen, bei Masthühnern waren dies 41,5 Tage. Damit gehörten beide Nutzungsarten zu Beginn der Erfassung zu den Nutzungsarten mit den höchsten populationsweiten Therapiehäufigkeiten. In den folgenden 10 Jahren waren in den beiden Mastgeflügelarten allerdings unterschiedliche Entwicklungen des Antibiotikaeinsatzes zu beobachten. Während sich der Einsatz bei Mastputen bis 2024 um etwa 26 % auf 45,6 Tage reduzierte (wenn auch mit zwischenzeitlichen Phasen des Anstieges), erhöhte sich der Einsatz bei Masthühnern nach anfänglichen Reduktionserfolgen wieder und liegt seit 2020 über dem Ausgangsniveau. Im Jahr 2024 lag die populationsweite Therapiehäufigkeit bei Masthühnern mit 45,9 Tagen um 11 % über der von 2014.

Auch die Wirkstoffklassenauswahl und ihre Entwicklung stellen sich zum Teil sehr unterschiedlich dar. Bei Mastputen spielen Penicilline die größte Rolle, wobei ihr Einsatz etwa proportional zur sinkenden Gesamttherapiehäufigkeit zurückging. Fluorchinolone und andere Chinolone wurden in 2024 um 35 % seltener eingesetzt als noch 2014. Beim Colistin betrug der Rückgang sogar 71 %. Damit wurde die Häufigkeit der Anwendung kritischer Antibiotika überproportional reduziert, wenngleich sie nach wie vor eine wichtige Rolle bei der antibiotischen Therapie spielen. Bei Masthühnern ist der Anstieg der populationsweiten Therapiehäufigkeit insgesamt vor allem auf den deutlich erhöhten Einsatz der 2 wichtigsten Wirkstoffklassen in dieser Nutzungsart zurückzuführen. Die Häufigkeit des Einsatzes stieg bei Lincosamiden von 2014 zu 2024 um 90 %, bei Aminoglykosiden um 78 %. Deutlich seltener als zu Beginn der Erfassung werden bei Masthühnern aber kritische Antibiotika eingesetzt: Die populationsweite Therapiehäufigkeit mit Fluorchinolonen nahm um 77 % ab, die mit Colistin um 64 %.

**Abb. 3:**
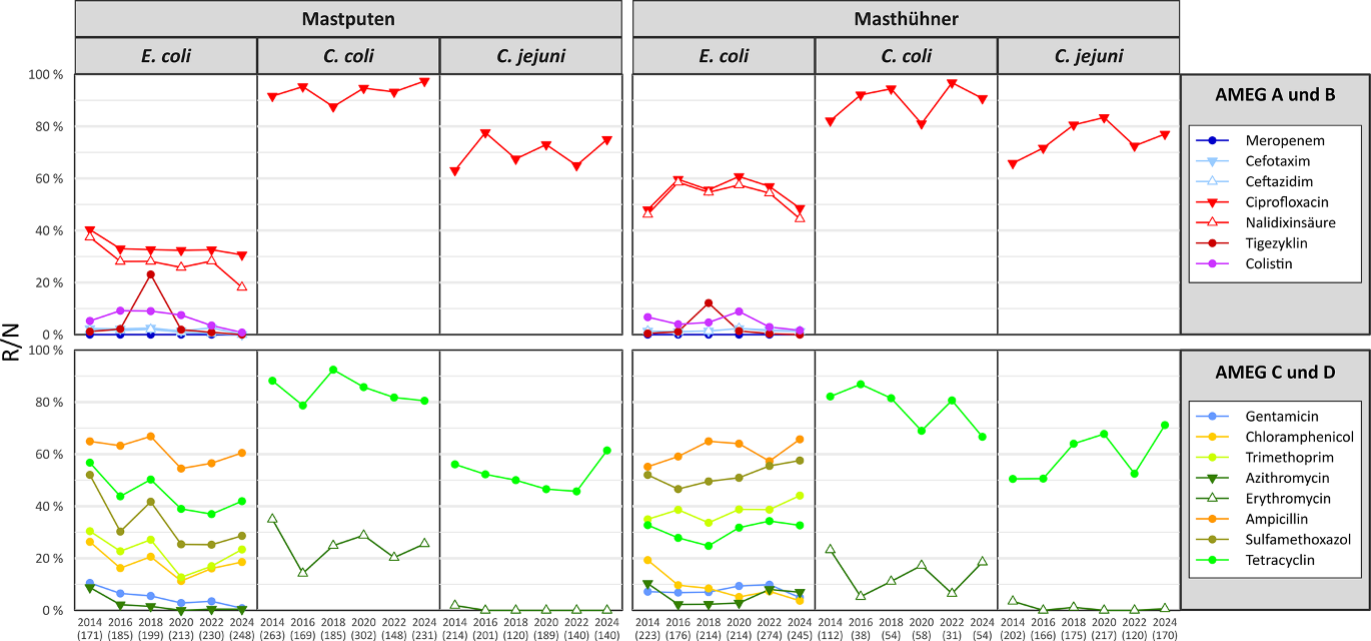
Resistenz von *E.* *coli,* *C.* *coli* und *C.* *jejuni* aus dem Blinddarminhalt von Mastputen und Masthühnern bei der Schlachtung, 2014 bis 2024. Obere Zeile: prozentuale Anteile resistenter (R) Isolate gegen kritische Wirkstoffe der AMEG-Kategorien A und B, untere Zeile: Anteile resistenter Isolate gegen weitere Wirkstoffe der AMEG-Kategorien C und D. Unter den Jahreszahlen ist in Klammern jeweils die Stichprobengröße (*N*) angegeben. *AMEG* Kategorien der „Antimicrobial Advice ad hoc Expert Group“ der Europäischen Arzneimittel-Agentur (EMA), *C.* *Campylobacter*, *E.* *Escherichia*

**Abb. 4:**
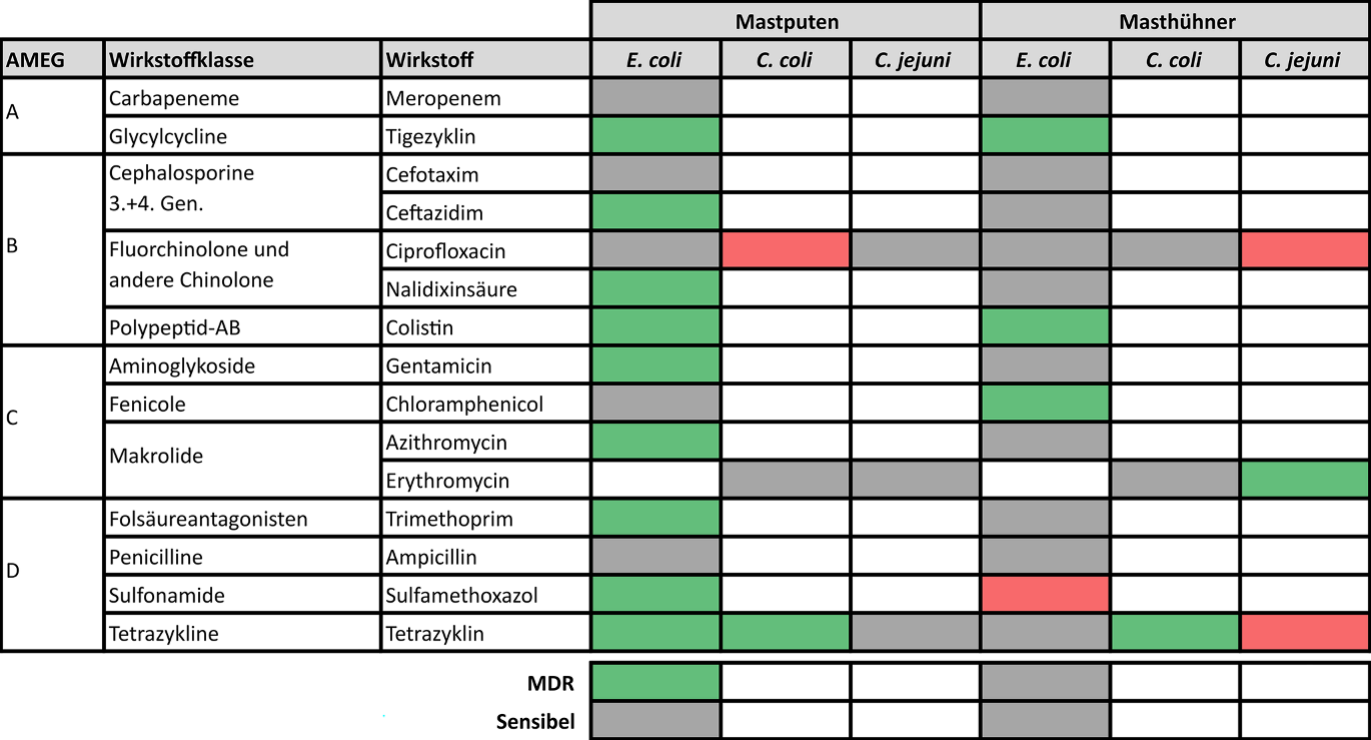
Trend der Resistenzraten von *E.* *coli,* *C.* *coli* und *C.* *jejuni* aus dem Blinddarminhalt von Mastputen und Masthühnern bei der Schlachtung von 2014 bis 2024. Farbkodierung in den Spalten „*E. coli*“, „*C. coli*“ und „*C. jejuni*“: Grün – statistisch signifikante Abnahme; *rot* – statistisch signifikante Zunahme; *grau* – Änderung nicht statistisch signifikant; *weiß* – nicht getestet/keine Daten. Für *Campylobacter* wird der Trend des Anteils sensibler Isolate und multiresistenter Isolate (MDR) nicht betrachtet, weil im Beobachtungszeitraum das Panel der getesteten Substanzen verändert wurde. *AB *Antibiotika*, AMEG* Kategorien der „Antimicrobial Advice ad hoc Expert Group“ der Europäischen Arzneimittel-Agentur (EMA), *C.* *Campylobacter*, *E.* *Escherichia*, *Gen.* Generation

Die Resistenzraten von *E. coli, C. coli* und *C. jejuni* aus dem Blinddarminhalt von Mastputen und Masthühnern bei der Schlachtung sind in Abb. 3 dargestellt, jeweils für den Zeitraum von 2014 bis 2024. *E. coli* von Mastputen wiesen in diesem Zeitraum statistisch signifikant sinkende Resistenzraten gegenüber Tetracyclin, Sulfamethoxazol, Trimethoprim, Gentamicin, Azithromycin, Tigecyclin, Nalidixinsäure, Ceftazidim und Colistin auf (Abb. 4). Gleichzeitig wurde bei keiner Substanz ein signifikanter Anstieg festgestellt. Entsprechend ging auch der Anteil der multiresistenten Isolate signifikant zurück. Allerdings wurden 2024 gegenüber 5 Substanzen numerisch höhere Resistenzraten beobachtet als 2022. Die Resistenz von *C. jejuni* gegenüber den 3 kontinuierlich getesteten Substanzen veränderte sich zwischen 2014 und 2024 nicht signifikant. Bei *C. coli* wurde gegenüber Ciprofloxacin eine signifikante Erhöhung der Resistenzrate gefunden.

Die Resistenz von *E. coli* bei Masthühnern zeigte zwischen 2014 und 2024 ungleichmäßige Veränderungen (Abb. 4). Während die Resistenz gegenüber Chloramphenicol, Tigecyclin und Colistin statistisch signifikant zurückging, stieg sie gegenüber Sulfamethoxazol statistisch signifikant an. Die Resistenz von *C. jejuni* aus Masthühnern gegenüber Tetracyclin und Ciprofloxacin stieg zwischen 2014 und 2024 signifikant an. Ein Rückgang wurde hier nur bei der ohnehin sehr seltenen Resistenz gegenüber Erythromycin beobachtet. Bei *C. coli* ergab sich im Gegensatz zu *C. jejuni *ein signifikanter Rückgang der Resistenz gegenüber Tetracyclin.

### Auswirkungen auf den Menschen

In dem JIACRA-Bericht zeigten die multivariaten Modelle deutliche Unterschiede zwischen *E. coli* und *C. jejuni*, was die statistischen Beziehungen zwischen dem Einsatz von Fluorchinolonen und der Resistenz der Mikroorganismen bei Tieren und den jeweiligen Werten beim Menschen betrifft (vgl. Abb. 37 in [19] für *E. coli* und Abb. 38 ebenda für *C. jejuni*). Für *E. coli* wurde sowohl bei Nutztieren als auch beim Menschen eine statistisch signifikante positive Beziehung zwischen dem Einsatz von Antibiotika und der Resistenz beobachtet. Der vermehrte Einsatz von Fluorchinolonen bei Tieren hing demnach mit höheren Resistenzraten von aus Tieren isolierten *E. coli* gegenüber Fluorchinolonen zusammen, dasselbe galt für Einsatz und Resistenz beim Menschen. Es gab jedoch keine Beziehung zwischen der Fluorchinolon-Resistenz bei *E.-coli*-Isolaten von Tieren und der bei Isolaten von Menschen.

Bei *C. jejuni* ergab sich ein anderes Bild: Hier zeigte sich zwar eine statistisch signifikante positive Beziehung zwischen dem Einsatz von Fluorchinolonen beim Geflügel und der Fluorchinolon-Resistenz von *C. jejuni* vom Geflügel. Eine solche Beziehung wurde jedoch nicht zwischen dem Einsatz von Fluorchinolonen beim Menschen und der Fluorchinolon-Resistenz von *C. jejuni* vom Menschen beobachtet. Allerdings gab es in diesem Fall eine klare statistisch signifikante positive Beziehung zwischen der Fluorchinolon-Resistenz von *C. jejuni* vom Geflügel und der Fluorchinolon-Resistenz von *C. jejuni* beim Menschen.

Dem Bericht ist zu entnehmen, dass die langfristige Entwicklung der Resistenz von *E. coli* zwischen 2014 und 2021 gegenüber Fluorchinolonen sowohl beim Menschen als auch bei Nutztieren rückläufig war (siehe Tab. 58 und Abbildungen 40 und 41 in [19]). Mehrere Staaten zeigten einen signifikanten Rückgang des Verbrauchs von Fluorchinolonen und der Resistenz von *E. coli* gegenüber Fluorchinolonen. Ein Anstieg beider Parameter wurde für keinen Staat beobachtet.

## Diskussion

Die Frage, welche Bedeutung die Resistenz von Bakterien bei Tieren für das Resistenzrisiko beim Menschen hat, ist eine der klassischen One-Health-Fragen. Über die möglichen Übertragungswege wurde bereits 2018 in dieser Zeitschrift berichtet [20] und an dieser Situation hat sich seitdem auch nichts grundsätzlich geändert. Zur Frage der Beziehung der Situation beim Tier zu der Resistenzsituation beim Menschen sind seither einige relevante Studien erschienen. Dabei sind mehrere Aspekte zu bedenken:

Eine Resistenzselektion durch den Einsatz von Antibiotika erfolgt sowohl bei Menschen als auch bei Tieren. Da oftmals auf dieselben Substanzklassen zurückgegriffen wird, ist es oft schwer zu unterscheiden, ob das Resistenzmuster in der Humanmedizin als Infektionserreger in Erscheinung tretender Bakterien eher durch den Antibiotikaeinsatz beim Menschen geformt wurde oder durch einen Übertrag aus der Tierhaltung, sei es durch Kontakt zu den Tieren, durch Lebensmittel oder die Exposition über die gemeinsame Umwelt. Dies gilt uneingeschränkt für die Resistenz von *E. coli*, da dieses Bakterium konsistent bei Menschen und allen warmblütigen Tieren als Darmbewohner vorkommt. *E. coli* ist daher ein guter Spiegel des Selektionsdrucks, unter dem die Darmflora steht. Hier dominiert der Effekt der Resistenzselektion innerhalb der Population über einen möglichen zusätzlichen Übertrag aus anderen Populationen, z. B. vom Tier auf den Menschen über Lebensmittel (siehe Abb. 37 in [19]). In Analogie dazu wurde auch gezeigt, dass der Hauptanteil von Cephalosporin-resistenten *E. coli* bei Menschen in den Niederlanden von anderen Menschen stammte und nicht vom Tier [21]. Auch zeigten klinische Isolate von *E. coli* vom Menschen größere Ähnlichkeiten mit anderen Isolaten vom Menschen als mit denen von Tieren, wenn man ihre Resistenzmuster betrachtet [22].

Bei *Campylobacter* verhält es sich anders, weil *Campylobacter* nicht Teil der normalen Darmflora des Menschen ist. Er wird vor allem im Rahmen von akuten Infektionen beim Menschen nachgewiesen. In der Konsequenz ist *Campylobacter* beim Menschen im Gegensatz zu *E. coli* nur selten einem Selektionsdruck ausgesetzt. Dies bestätigt das in dieser Arbeit betrachtete Beispiel der Resistenz von *C. jejuni* gegen Fluorchinolone, die keine statistische Beziehung zum Einsatz von Fluorchinolonen beim Menschen zeigt (siehe Abb. 38 in [19]).

Die Übertragung vom Tier auf den Menschen kann nicht nur durch Lebensmittel, sondern auch durch Kontakt erfolgen. Dies ist für Staphylokokken gut belegt, bei denen in der Nutztierhaltung Beschäftigte ein deutlich höheres Risiko tragen, mit nutztierassoziierten Methicillin-resistenten *Staphylococcus aureus* (MRSA) besiedelt zu sein, als nicht beruflich exponierte Personen [23–26]. Assoziationen bestimmter Berufsgruppen konnten auch für Cephalosporin-resistente *E. coli* gezeigt werden. Hier wiesen die Bakterien von Nutztieren und ihren Haltern größere Ähnlichkeiten auf als zwischen Nutztieren und der Allgemeinbevölkerung [27]. Auch im Hinblick auf *Campylobacter*-Infektionen erhöhte der Kontakt zu Geflügel das Risiko einer Infektion deutlich [28]. Bei *Campylobacter* konnte jedoch auch gezeigt werden, dass ein großer Anteil der Infektionen des Menschen mit Isolaten assoziiert ist, die wahrscheinlich vom Geflügel stammen, und dass der Verzehr von Geflügelfleisch hier eine Rolle spielt [28, 29].

Insgesamt haben sich in Deutschland die Abgabemengen von Antibiotika in der Tierhaltung in den letzten 10 Jahren deutlich verringert [30]. Eine nähere Analyse der Daten zeigt aber, dass die Therapiehäufigkeit sich nicht in allen Nutztierpopulationen gleichsinnig entwickelt hat. So wurden bei Rindern, Schweinen und Puten Reduktionen beobachtet, während dies für Masthühner nur temporär der Fall war. Die Betrachtung der Resistenzergebnisse von *E. coli* von Schlachttieren zeigt, dass sich dies auch auf die Exposition des Menschen gegenüber resistenten Mikroorganismen auswirkt. Die Resistenz bei Isolaten von *E. coli* aus dem Blinddarm von Puten bei der Schlachtung für 9 der 14 getesteten Substanzen sank insgesamt signifikant und entsprechend ging auch der Anteil multiresistenter Isolate zurück. Dass 2024 gegenüber 5 Substanzen numerisch höhere Resistenzraten beobachtet wurden als noch 2022, könnte allerdings darauf hindeuten, dass der Rückgang der Resistenzraten sich nicht so fortsetzt wie in den Jahren zuvor. Dies ist angesichts der seit 2022 wieder ansteigenden Therapiehäufigkeiten bei Mastputen mit Sorge zu betrachten. Bei Isolaten von Masthühnern wurden unterschiedliche Entwicklungen beobachtet. Hier sank die Resistenzrate bei Isolaten von Schlachttieren nur gegenüber solchen Substanzen, die entweder nicht eingesetzt wurden (Chloramphenicol, Tigecyclin) oder aber einen deutlich reduzierten Einsatz zeigten (Colistin), während die Resistenzrate gegen Sulfamethoxazol sogar stieg. Dies zeigt, dass eine konsequente Reduktion der Häufigkeit, mit der Tiere mit Antibiotika behandelt werden, zu einer Verbesserung der Resistenzsituation von Bakterien bei diesen Tieren beitragen kann. Dies wurde auch in Studien in mehreren anderen Ländern gezeigt, die mit anderen Konzepten ebenfalls eine Reduktion erreichten [27, 31]. Es zeigt auch, dass das 2014 eingeführte Antibiotikaminimierungskonzept in weiten Teilen im Sinne einer Reduktion des Antibiotikaeinsatzes funktioniert hat, dass es aber auch Bereiche gibt, in denen keine Reduktion erzielt wurde. Diese sollten künftig verstärkt in den Fokus genommen werden.

Etwas weniger optimistisch stimmen die Ergebnisse der Resistenzuntersuchungen bei *C. coli* und *C. jejuni *aus Blinddarmproben von Tieren bei der Schlachtung. Bei diesen hat sich die erreichte Reduktion der Therapiehäufigkeit bei Puten bisher nicht in signifikant verringerten Resistenzraten niedergeschlagen. Die Resistenzraten von *C. coli* gegenüber Ciprofloxacin bewegen sich sowohl bei Mastputen als auch Masthühnern auf einem sehr hohen Niveau, stiegen jedoch im betrachteten 10-Jahres-Zeitraum sogar an. Bei den Masthühnern kam es zu einem Anstieg der Resistenz von *C. jejuni* gegenüber Ciprofloxacin und Tetracyclin. Hier sank die Resistenzrate gegenüber Erythromycin bei *C. jejuni* und gegenüber Tetracyclin bei *C. coli*. Allerdings bewegte sich die Resistenzrate gegenüber Erythromycin immer schon auf einem sehr niedrigen Niveau. Insgesamt ist die Entwicklung also auch hier bei den Masthühnern weniger günstig als bei den Puten. Warum die Resistenzentwicklung bei *Campylobacter* insgesamt weniger günstig ist als bei *E. coli*, ist nicht bekannt und bedarf weiterer Untersuchungen. Für die Resistenz von *C. jejuni* gegen das Fluorchinolon Ciprofloxacin konnte gezeigt werden, dass diese sich in den letzten Jahren vergleichsweise unabhängig vom Einsatz von Fluorchinolonen entwickelt hat. Hintergrund scheint zu sein, dass die Resistenz vermittelnde Eigenschaft genetisch an ein Fitness-assoziiertes Gen gekoppelt ist und sich dadurch auch unabhängig vom Fluorchinoloneinsatz vermehren kann [32]. Die weniger günstige Resistenzentwicklung wiegt umso schwerer, als *Campylobacter* beim Menschen die häufigsten Erreger bakterieller Darmerkrankungen in Europa sind und die resistenten Mikroorganismen unmittelbar vom Tier über das Lebensmittel auf den Menschen übertragen werden [28, 29]. Hier gibt es also eine unmittelbare Beziehung zwischen Erregerübertragung und Erkrankung. Damit wird hier die beim Tier erzeugte Resistenzselektion unmittelbar klinisch für den Menschen bedeutsam. Um die Exposition der Verbraucherinnen und Verbraucher mit resistenten *Campylobacter* zu reduzieren, ist es dringend notwendig, die Resistenzraten von *Campylobacter* bei Nutztieren zu verringern sowie die Übertragung dieser Bakterien auf die Schlachtkörper vor allem von Masthühnern effektiver zu unterbinden. Mit einer sehr guten Küchenhygiene lassen sich zwar viele der *Campylobacter*-Infektionen vermeiden, aber die Zahl auftretender Infektionen zeigt, dass es hier nach wie vor Verbesserungspotenzial gibt.

## Fazit

Der Reduktion des Antibiotikaeinsatzes in der Tierhaltung in Deutschland folgte in weiten Teilen ein signifikanter Rückgang der Resistenzraten von *E. coli* von diesen Tieren, in der vorliegenden Arbeit beispielhaft anhand der Mastputen dargestellt. Eine Ausnahme stellen Masthühner und das Hähnchenfleisch dar, bei denen es zu keiner nachhaltigen Reduktion des Antibiotikaeinsatzes kam und folgerichtig die Resistenzraten auch nicht sanken. Sinkende Resistenzraten bedeuten, wenn es denn zu einer Übertragung der Bakterien auf den Menschen kommt, eine Exposition gegenüber weniger resistenten Bakterien. Das heißt, etwaige Infektionen mit diesen Erregern sind weniger schwer zu therapieren und das Risiko einer horizontalen Übertragung von Resistenzgenen auf die Mikroflora des Menschen sinkt.

Bei *Campylobacter* trat keine so deutliche Reduktion der Resistenzraten ein. Die Ursache für diese Diskrepanz ist nicht in jedem Fall klar. Für einzelne Resistenzen, etwa gegenüber dem Fluorchinolon Ciprofloxacin, ist beschrieben, dass die Resistenz genetisch an einen weiteren Fitnessfaktor für *C. jejuni* gekoppelt ist, sodass sie auch weiter steigen kann, wenn die Substanz nicht eingesetzt wird.

### Infobox 1: Therapiehäufigkeit (TH)

Betriebliche TH

- Maß für den Antibiotikaeinsatz auf Betriebsebene
- Berechnung je Betrieb, Nutzungsart und Halbjahr
- Grundlage des betrieblichen Benchmarkingsystems
- Gibt an, an wie vielen Tagen des Halbjahres ein im Betrieb durchschnittlich gehaltenes Tier antibiotisch behandelt wurde

Populationsweite TH

- Maß für den Antibiotikaeinsatz in der durchschnittlichen Gesamtpopulation einer Nutzungsart, d. h. über alle Betriebe in Deutschland hinweg
- Berechnung je Nutzungsart und Jahr
- Stratifizierung nach Wirkstoffklasse
- Gibt an, an wie vielen Tagen des Jahres ein Tier der durchschnittlichen Gesamtpopulation antibiotisch behandelt wurde

### Infobox 2: Kategorien für die sorgfältige und verantwortungsvolle Anwendung von Antibiotika in der Veterinärmedizin. Erstellt von der Antimicrobial Advice ad hoc Expert Group (AMEG) der Europäischen Arzneimittel-Agentur (EMA; [8])

- A (Vermeiden): der Humanmedizin vorbehalten; dürfen bei Lebensmittel liefernden Tieren nicht angewendet werden
- B (Beschränken): von kritischer Bedeutung für die Humanmedizin; Anwendung nur, wenn keine Alternativen aus C oder D verfügbar
- C (Vorsicht): es gibt in der Humanmedizin Alternativen; Anwendung nur, wenn keine Alternativen aus D verfügbar
- D (Sorgfalt): Erstlinientherapie

## Data Availability

Die Daten zum Antibiotikaeinsatz in der Nutztierhaltung sind in aggregierter Form in den Berichten des Bundesinstituts für Risikobewertung veröffentlicht [9–13]. Die Daten zur Antibiotikaresistenz stehen im Portal https://zoonotify.bfr.berlin zur Verfügung [15].

## Footnotes

1 Cephalosporine der 3. und 4. Generation sind allerdings nicht für die Anwendung bei Geflügel zugelassen und werden demzufolge auch nicht eingesetzt.

## References

[1] Bundesministerium für Ernährung und Landwirtschaft (2019) Bericht des Bundesministeriums für Ernährung und Landwirtschaft über die Evaluierung des Antibiotikaminimierungskonzepts der 16. AMG-Novelle. https://www.bmleh.de/DE/themen/tiere/tierarzneimittel/kurzfassung16-amg-novelle.html. Zugegriffen: 21.01.2026

[2] Bundesinstitut für Risikobewertung (2026) Antibiotika-Einsatz bei Nutztieren. https://www.bfr.bund.de/lebensmittel-und-futtermittelsicherheit/bewertung-mikrobieller-risiken-von-lebensmitteln/antibiotika-einsatz-bei-nutztieren/. Zugegriffen: 26.01.2026

[3] European Medicines Agency (2026) Analysis of antimicrobial consumption and resistance (’JIACRA’ reports). https://www.ema.europa.eu/en/veterinary-regulatory-overview/antimicrobial-resistance-veterinary-medicine/analysis-antimicrobial-consumption-resistance-jiacra-reports. Zugegriffen: 26.01.2026

[4] 16. AMG-Novelle (2013) Sechzehntes Gesetz zur Änderung des Arzneimittelgesetzes. Gesetz vom 10.10.2013 (BGBl. I S. 3813, 2014 I 272). https://www.buzer.de/gesetz/10963/index.htm. Zugegriffen: 21.01.2026

[5] 17. AMG-Novelle (2021) Siebzehntes Gesetz zur Änderung des Arzneimittelgesetzes. Gesetz vom 10.08.2021 (BGBl. I S. 3519, Nr. 53). https://www.buzer.de/gesetz/14907/index.htm. Zugegriffen: 21.01.2026

[6] TAMG (2021) Gesetz über den Verkehr mit Tierarzneimitteln und zur Durchführung unionsrechtlicher Vorschriften betreffend Tierarzneimittel (Tierarzneimittelgesetz - TAMG). Gesetz vom 27.09.2021 (BGBl. I S. 4530, Nr. 70). https://www.buzer.de/TAMG.htm. Zugegriffen: 26.01.2026

[7] TAMG-Novelle (2022) Gesetz zur Änderung des Tierarzneimittelgesetzes zur Erhebung von Daten über antibiotisch wirksame Arzneimittel und zur Änderung weiterer Vorschriften. Vorschrift vom 21.12.2022 (BGBl. I S. 2852, Nr. 57). https://www.buzer.de/gesetz/15684/index.htm. Zugegriffen: 20.01.2026

[8] European Medicines Agency (2019) Categorisation of antibiotics in the European Union. European Medicines Agency (EMA), Committee for Medicinal Products for Veterinary Use (CVMP), Committee for Medicinal Products for Human Use (CHMP), EMA/CVMP/CHMP/682198/2017, https://www.ema.europa.eu/en/documents/report/categorisation-antibiotics-european-union-answer-request-european-commission-updating-scientific_en.pdf. Zugegriffen: 21.01.2026

[9] Flor M, Käsbohrer A, Kaspar H, Tenhagen B‑A, Wallmann J (2019) Beiträge der Arbeitsgruppe Antibiotikaresistenz des Bundesinstituts für Risikobewertung (BfR) und des Bundesamtes für Verbraucherschutz und Lebensmittelsicherheit (BVL) zur Evaluierung der 16. AMG-Novelle. Themenkomplex 1: Entwicklung der Antibiotikaabgabe- und -verbrauchsmengen sowie der Therapiehäufigkeit. https://www.bmleh.de/SharedDocs/Downloads/DE/_Tiere/Tiergesundheit/Tierarzneimittel/16-AMG-Novelle-Anlage2.pdf?__blob=publicationFile&v=2. Zugegriffen: 21.01.2026

[10] Flor M, Käsbohrer A, Tenhagen B‑A (2022) Therapiehäufigkeit und Antibiotikaverbrauchsmengen 2018–2021: Entwicklung in zur Fleischerzeugung gehaltenen Rindern, Schweinen, Hühnern und Puten. Bundesinstitut für Risikobewertung (Hrsg) Bericht vom 20. Dezember 2022. 10.17590/20221216-083830. Zugegriffen: 21.01.2026

[11] Flor M, Käsbohrer A, Tenhagen B‑A (2023) Therapiehäufigkeit und Antibiotika-Verbrauchsmengen 2022: Entwicklung in zur Fleischerzeugung gehaltenen Rindern, Schweinen, Hühnern und Puten. Bundesinstitut für Risikobewertung (Hrsg) Wissenschaftsbericht vom 31. August 2023. 10.17590/20230831-091916-0. Zugegriffen: 21.01.2026

[12] Flor M, Käsbohrer A, Tenhagen B‑A (2024) Antibiotika-Verbrauchsmengen und Therapiehäufigkeit 2023: Entwicklung in Rindern, Schweinen, Hühnern und Puten. Bundesinstitut für Risikobewertung (Hrsg) Wissenschaftsbericht des BfR vom 30. August 2024. 10.17590/20240830-135348-0. Zugegriffen: 21.01.2026

[13] Flor M, Tenhagen B‑A (2025) Antibiotika-Verbrauchsmengen und Therapiehäufigkeit 2024: Entwicklung in Rindern, Schweinen, Hühnern und Puten. Bundesinstitut für Risikobewertung (Hrsg) Wissenschaftsbericht des BfR vom 28. August 2025. 10.17590/20250825-094624-0. Zugegriffen: 21.01.2026

[14] Bundesamt für Verbraucherschutz und Lebensmittelsicherheit (2026) Zoonosen-Monitoring. https://www.bvl.bund.de/DE/Arbeitsbereiche/01_Lebensmittel/01_Aufgaben/02_AmtlicheLebensmittelueberwachung/06_ZoonosenMonitoring/lm_zoonosen_monitoring_node.html. Zugegriffen: 26.01.2026

[15] Bundesinstitut für Risikobewertung (2026) ZooNotify. https://zoonotify.bfr.berlin. Zugegriffen: 16.03.2026

[16] European Centre for Disease Prevention and Control (ECDC), European Food Safety Authority (EFSA), European Medicines Agency (EMA) (2015) First joint report on the integrated analysis of the consumption of antimicrobial agents and occurrence of antimicrobial resistance in bacteria from humans and food-producing animals, JIACRA, 2011–2012. EFSA J. 13:114. 10.2903/j.efsa.2015.4006

[17] European Centre for Disease Prevention and Control (ECDC), European Food Safety Authority (EFSA), European Medicines Agency (EMA) (2017) Second joint report on the integrated analysis of the consumption of antimicrobial agents and occurrence of antimicrobial resistance in bacteria from humans and food-producing animals. JIACRA II, 2013–2015. EFSA J. 15:4872. 10.2903/j.efsa.2017.4872 10.2903/j.efsa.2017.4872 PMC7009874 32625542

[18] European Centre for Disease Prevention and Control (ECDC), European Food Safety Authority (EFSA), European Medicines Agency (EMA) (2021) Third joint inter-agency report on integrated analysis of consumption of antimicrobial agents and occurrence of antimicrobial resistance in bacteria from humans and food-producing animals in the EU/EEA, JIACRA III, 2016–2018. EFSA J. 19:e06712. 10.2903/j.efsa.2021.6712 10.2903/j.efsa.2021.6712 PMC8243991 34221148

[19] European Centre for Disease Prevention and Control (ECDC), European Food Safety Authority (EFSA), European Medicines Agency (EMA) (2024) Fourth joint inter-agency report on integrated analysis of consumption of antimicrobial agents and occurrence of antimicrobial resistance in bacteria from humans and food-producing animals in the EU/EEA, JIACRA IV, 2019–2021. EFSA J. 22:166. 10.2903/j.efsa.2024.8589 10.2903/j.efsa.2024.8589 PMC10885775 38405113

[20] Tenhagen B‑A, Werner N, Kasbohrer A, Kreienbrock L (2018) Transmission pathways for resistant bacteria between animals and humans: antibiotics resistance in the One Health context. Bundesgesundheitsblatt 61:515–521. 10.1007/s00103-018-2717-z 10.1007/s00103-018-2717-z 29616289

[21] Mughini-Gras L, Dorado-García A, van Duijkeren E et al. (2019) Attributable sources of community-acquired carriage of containing β‑lactam antibiotic resistance genes: a population-based modelling study. Lancet Planet Health 3:E357-E369. 10.1016/S2542-5196(19)30130-5 10.1016/S2542-5196(19)30130-5 31439317

[22] Suwono B, Eckmanns T, Kaspar H et al. (2021) Cluster analysis of resistance combinations in from different human and animal populations in Germany 2014-2017. PLoS One 16:e0244413. 10.1371/journal.pone.0244413 10.1371/journal.pone.0244413 PMC7817003 33471826

[23] Köck R, Brase K, Harlizius J et al. (2013) MRSA and ESBL-producing enterobacteria in German pig holdings. Int. J. Med. Microbiol. 303:108–108. 10.1016/j.ijmm.2013.08.004

[24] Bisdorff B, Scholhölter JL, Claussen K, Pulz M, Nowak D, Radon K (2012) MRSA-ST398 in livestock farmers and neighbouring residents in a rural area in Germany. Epidemiology and Infection 140:1800–1808. 10.1017/S0950268811002378 10.1017/S0950268811002378 22313681

[25] Mulders MN, Haenen APJ, Geenen PL et al. (2010) Prevalence of livestock-associated MRSA in broiler flocks and risk factors for slaughterhouse personnel in The Netherlands. Epidemiol. Infect. 138:743–755. 10.1017/S0950268810000075 10.1017/S0950268810000075 20109255

[26] van Cleef BAGL, Broens EM, Voss A et al. (2010) High prevalence of nasal MRSA carriage in slaughterhouse workers in contact with live pigs in The Netherlands. Epidemiol. Infect. 138:756–763. 10.1017/S0950268810000245 10.1017/S0950268810000245 20141647

[27] Dorado-Garcia A, Mevius DJ, Jacobs JJ et al. (2016) Quantitative assessment of antimicrobial resistance in livestock during the course of a nationwide antimicrobial use reduction in the Netherlands. J. Antimicrob. Chemother. 71:3607–3619. 10.1093/jac/dkw308 10.1093/jac/dkw308 27585970

[28] Rosner BM, Schielke A, Didelot X et al. (2017) A combined case-control and molecular source attribution study of human infections in Germany, 2011–2014. Sci. Rep. 7:5139. 10.1038/s41598-017-05227-x 10.1038/s41598-017-05227-x PMC5505968 28698561

[29] Pascoe B, Futcher G, Pensar J et al. (2024) Machine learning to attribute the source of infections in the United States: A retrospective analysis of national surveillance data. J. Infect. 89:106265. 10.1016/j.jinf.2024.106265 10.1016/j.jinf.2024.106265 PMC7617841 39245152

[30] Siller P, Klabunde-Negatsch A, Sander S, Heberer T (2024) Abgabemengenerfassung von Antibiotika in Deutschland 2023. Deutsches Tierärzteblatt 72:1554–1567.

[31] Perrin-Guyomard A, Houée P, Lucas P et al. (2023) Prevalence and molecular epidemiology of mcr-mediated colistin-resistance Escherichia coli from healthy poultry in France after national plan to reduce exposure to colistin in farm. Front. Microbiol. 14:1254122. 10.3389/fmicb.2023.1254122 10.3389/fmicb.2023.1254122 PMC10587439 37869671

[32] Luo ND, Pereira S, Sahin O et al. (2005) Enhanced fitness of fluoroquinolone-resistant in the absence of antibiotic selection pressure. Proc. Natl. Acad. Sci. U. S. A. 102:541–546. 10.1073/pnas.0408966102. 10.1073/pnas.0408966102 PMC545549 15634738

